# Effect of Grape Seed Proanthocyanidins on Fat Metabolism and Adipocytokines in Obese Rats

**DOI:** 10.3390/metabo13040568

**Published:** 2023-04-17

**Authors:** Pengxiang Gao, Luoyun Fang, Yucong Pan, Linshu Jiang

**Affiliations:** Department of Animal Science, Animal Science and Technology College, Beijing University of Agriculture, Beijing 102206, Chinajls@bua.edu.cn (L.J.)

**Keywords:** polyphenols, lipids metabolisms, 3T3-L1 preadipocytes, adipocytokines

## Abstract

This study aimed to investigate the effect of Grape Seed Proanthocyanidin (GSP) on fat metabolism and adipocytokines in obese rats. Fifty 5-week-old rats were randomly assigned to five groups (*n* = 10 per group) and given either a basal diet, a high-fat diet, or a high-fat diet supplemented with GSP (25, 50, and 100 mg/d) per group. The experiment lasted for five weeks, including a one-week adaptation period and a four-week treatment period. At the end of the experimental period, serum and adipose tissue samples were collected and analyzed. Additionally, we co-cultured 3T3-L1 preadipocytes with varying concentrations of GSP to explore its effect on adipocyte metabolism. The results demonstrated that GSP supplementation reduced weight, daily gain, and abdominal fat weight coefficient (*p* < 0.05). It also decreased levels of glucose, cholesterol (TC) (*p* < 0.05), triglycerides (TG) (*p* < 0.05), low-density lipoprotein (LDL), cyclooxygenase-2 (COX-2), and interleukin-6 (IL-6) in adipose tissue. Furthermore, GSP addition caused adipocyte crumpling in vitro and reduced the mRNA expression of COX-2, LEP, and TNF-α in adipocytes in vitro. These findings provide compelling evidence for exploring the role of GSP in the prevention and treatment of obesity and related diseases.

## 1. Introduction

Grape seed proanthocyanidin (GSP) is a natural mixture derived from the seeds of *Vitis vinifera* [1]. To obtain GSP, the grape seeds were air-dried for a week, ground into a fine powder, and then macerated in 70% ethanol (25% *w*/*v*) for 3 days at room temperature before being filtered [2]. Dried GSP has been found to contain high levels of total phenolic content (16.2 g/kg), condensed tannins (515.1 mg/kg), and soluble (219.1 g/kg) [3]. The role of GSP in defending organs and tissues against obesity, hyperlipidemia, inflammation, and oxidative stress has been widely acknowledged [4]. Additionally, dietary supplementation with GSP has been shown to repress fatty liver disease, decrease liver size, and alleviate the level of steatosis in hyperlipidemic rats, suggesting that GSP may be an effective therapy for obesity-related diseases in animal models.

Obesity is a global health problem that has been extensively studied. Epidemiological and clinical research has shown that obesity can lead to a number of dangerous diseases, including hypertension, cardiovascular disease, diabetes, and atherosclerosis, due to its effects on blood pressure, blood glucose, and blood lipid levels [5,6]. These metabolic changes are closely associated with the development of obesity, diabetes type-2, abnormal lipid metabolism, and cardiovascular disease [7,8]. The white adipose tissue (WAT) is the principal organ affected in obesity, and it undergoes hypertrophy and hyperplasia due to the incorporation of triglycerides (TAG) into its cytoplasm [9]. Lipopolysaccharides (LPS) is an endotoxin from Gram-negative gut bacteria that triggers the activation of the Toll-like receptor-4 (TLR-4) inflammatory pathway and can modulate WAT metabolism [10]. Yasmin et al. [11] have found that grape seed polyphenols (GSP) can improve intestinal permeability and microbial diversity, and reduce LPS levels by down-regulating the TLR-4 pathway, thus preventing WAT hypertrophy. In addition, GSP has been shown to reduce the number of adipocytes [12]. Therefore, GSP holds great promise as a treatment against obesity. In conclusion, the findings of this study suggest that GSP may be an effective treatment for obesity. Further research is needed to fully understand the mechanism of action of GSP and to determine its potential use in clinical settings.

Adipocytokines refer to a group of cytokines expressed and secreted by adipocytes, including both adipocyte-specific and non-adipocyte-specific cytokines. Numerous studies have demonstrated that the dysregulation of adipocytokine secretion can directly contribute to the development of obesity [13]. Cytokines are involved in the regulation of adipocyte proliferation and differentiation, and abnormal adipocytokine secretion and dysfunction are commonly observed in obese individuals. Therefore, restoring normal adipocyte expression and function is a crucial component of the prevention and treatment of obesity and related diseases.

3T3-L1 preadipocytes are widely used to model adipocytes, as they can be differentiated into adipocytes under appropriate conditions [14]. Hongyun Yan et al. [15] have reported that COX-2 is implicated in body fat regulation, as rats heterozygous for the COX-2 gene showed a 30% increase in body weight. COX-2 expression is primarily regulated at the transcriptional level, predominantly via the NF-κB pathway. Notably, PPARγ, the key regulator of adipocyte differentiation, suppresses the NF-κB pathway.

To date, there have been limited studies on the role of GSP in adipogenesis and its underlying mechanism remains unclear. Here, we hypothesize that dietary supplementation of GSP may increase COX-2 expression and affect inflammatory markers, such as IL-6 and LEP. We used proanthocyanidins, a natural plant extract, to investigate the effects of proanthocyanidins on fat metabolism and adipocytokines in obese rats and to develop proanthocyanidins as a functional feed additive via dietary regulation. This study aims to evaluate the feasibility of using GSP as a functional food to combat obesity.

## 2. Materials and Methods

### 2.1. Sources

In this study, GSP with a purity greater than 70% (with other sugars including fructose and sucrose) in powder form was used, extracted from JF-NATURAL (Tianjin, China).

Standard diets with the following specifications were used: moisture content less than or equal to 10%, crude protein content greater than or equal to 20%, crude ash content less than or equal to 8%, crude fiber content less than or equal to 5%, calcium content between 1.8% and 1%, total phosphorus content between 0.6% and 1.2%, lysine content greater than or equal to 1.35%, and salt content of 0.5%.

High-fat diets, which included 10% egg yolk powder, 10% lard, 1% cholesterol, and 79% standard diet, were purchased from Beijing Keao Xieli Feed Co (Beijing Keao Xieli Feed Co., Beijing, China).

### 2.2. Experimental Animals, Diets and Design

This study was performed in strict accordance with the China Laboratory Animal Welfare and Ethics Committee Guidelines (publication no. GB/T 35892-2018). All procedures conducted in the study were approved by the Animal Care Committee of Beijing University of Agriculture (Beijing, China).

The experiment was conducted at the Beijing Key Laboratory of Dairy Cow Nutrition and lasted for 5 weeks, including 1 week of adaptation and 4 weeks of treatment, from mid-May to late June. Fifty rats with a similar age of 5 weeks were randomly divided into five groups: (A) standard diet group, (B) high-fat diet group, (C) high-fat diet + GSP 25 mg/d group, (D) high-fat diet + GSP 50 mg/d group, and (E) high-fat diet + GSP 100 mg/d group. Proanthocyanidins were dissolved in water according to the rats’ water consumption during the adaptation period. Rats were provided with food and water ad libitum after administration.

Cages were disinfected with 0.1% potassium permanganate, washed with water, and set aside before the experiment. Bedding was changed once a week, and the diet was maintained throughout the study.

### 2.3. Sample Collection and Analyses

#### 2.3.1. Determination of Routine Indices in Rats

The initial body weight and daily weight changes of each rat were recorded. The blood of each rat was collected from a cardiac vein, and the levels of triglyceride, blood glucose, high-density lipoprotein (HDL), low-density lipoprotein (LDL), and cholesterol were determined using an automatic biochemical analyzer (7020 HITACHI, Tokyo, Japan). After blood centrifugation, serum was collected and growth hormone (ELISA kit R&D Systems, Minneapolis, MN, USA) and insulin (ELISA kit R&D Systems, Minneapolis, MN, USA) levels were determined according to the kit. The rats were weighed before they were killed, and after they were killed, the abdominal fat of the rats was removed and weighed. According to the formula: abdominal fat weight coefficient% = abdominal fat weight/body weight.

#### 2.3.2. Detection of Cytokines and mRNA Expression in Rat Adipose Tissue

We took rat adipose tissue, opened the rat abdominal cavity, removed rat abdominal adipose tissue with an aseptic scalpel, rinsed it with aseptic saline, cut it into uniform size with scissors and placed it in a frozen tube, froze it in a liquid nitrogen tank and stored it in a −80 °C refrigerator for testing. We took out the adipose tissue, cut it into about 1 g pieces, weighed it, ground it thoroughly with a mortar, added 20 times its weight-to-volume of normal saline, centrifuged samples for 10 min at 3000 rmp, collected the supernatant, put it into cryopreservation, and then the levels of COX-2, TNF-α, leptin, adiponectin, IL-6 and cAMP in the adipose tissue of rats were detected by kits (ELISA kit R&D Systems, Minneapolis, MN, USA).

#### 2.3.3. RNA Extraction and Quantitative Real-Time Polymerase Chain Reaction (qRT-PCR)

Total RNA was extracted from cells using Trizol (Invitrogen, Waltham, MA, USA) according to the manufacturer’s instructions. cDNA synthesis was performed using a Reverse Transcription Kit (Takara Bio Inc, Yamanashi, Japan). The sequence of primers was as follows: COX-2 sense (5′-CACGGACTTGCTCACTTTGT-3′), antisense (5′-GCCTTTGCCACTGCTTGTA-3′); leptin sense (5′-TTGGACCTTAGCCCTGAATG-3′), antisense (5′-CTGGATGACGGGTGTGAAC-3′); TNF-α sense (5′-GGTTCCGTCCCTCTCATACA-3′), antisense (5′-AGACACCGCCTGGAGTTCT-3′); β-actin sense (5′-CCCATCTATGAGGGTTACGC-3′), antisense (5′-TTTAATGTCACGCACGATTTC-3′).

#### 2.3.4. Effect of Procyanidins on the Proliferation of 3T3-L1 Preadipocytes

The experiment was divided into 1 control group and 9 experimental groups. The mixed cell suspension was added every 100 μL to a 96-well cell culture plate and the rim was filled with PBS. Cells were routinely cultured at 37 °C in a 5% CO_2_ incubator, with 5 replicate wells in each group. After the cells were attached to the wall, different concentrations of GSP storage solution were added to the experimental group, and the final concentrations were 0 (control), 25, 50, 100, 150, 200, 400, and 600 μg/mL, respectively, and cultured in a 37 ℃ and 5%CO_2_ incubator. After 24 h, 48 h, and 72 h of culture, the culture medium was discarded, washed twice with PBS, 100 μL complete medium was added, and then 20 μL MTT and 5 mg/mL were added in turn. After 4 h of culture, the supernatant was carefully aspirated, and 150 μL dimethyl sulfoxide (DMSO) was added. After mixing for 10 min, the OD490 nm value of each well was determined by enzyme-linked immunosorbent assay.

#### 2.3.5. Effect of Proanthocyanidins on Cytokines in Mature Adipocytes

The differentiation liquid A was prepared on the super clean table: a centrifuge tube of 50 mL was taken, the culture medium of 40 mL was added, and then 80 μLof IBMX storage solution, 40 μLof DSMS storage solution, and 40 μLof INS storage solution were mixed to form the adipocyte differentiation liquid A. (2) The old culture medium was absorbed from the culture flask, the differentiation medium A3ml was taken and added to the culture flask, and the cells were cultured for 48 h. (3) The differentiation medium B was distributed: we took the 50 mL centrifuge tube, added 20 mL of the culture medium, then added 20 μL of INS storage solution and mixed well, to prepare the adipocyte differentiation medium B. (4) The old culture medium from the culture flask was absorbed, 3 mL of the differentiation medium B was added to each culture flask, and the cells were cultured for 48 h. (5) We kept the culture in DMEM with 10% FBS and observed that the old medium became yellow and sticky and lipid droplets could be seen under the microscope. After 10–12 days of culture, differentiation was complete. (6) After the completion of cell differentiation, many lipid droplets were observed in the cells, and about 90% of the pre-3T3-L1 cells showed adipocyte phenotype 10 days later—the cells on the 14th day after differentiation were induced to carry out the following experiments.

Through the experiment, it was found that the optimal co-culture concentrations were 100, 150, and 200. The differentiated and mature adipocytes were co-cultured with GSP storage solution at the final concentrations of 0, 100, 150, and 200 μg/mL, with 3 replicates in each group, and cultured at 37 °C and 5% CO_2_ incubator, respectively. After 12, 24, and 48 h of culture, the culture supernatant and cells were collected by centrifugation. One milliliter of TRIZOL was added to each group of precipitated cells and stored at −80 °C for detection. The levels of the cytokines COX-2, TNF-α, leptin, adiponectin, IL-6, and cAMP in the supernatant were detected by the double antibody sandwich ELISA method. Triazole samples were taken from the refrigerator at −80 °C and dissolved on ice. The effect of proanthocyanidins on the expression of cytokine mRNA in mature adipocytes was determined according to the previous procedure.

#### 2.3.6. Statistical Analysis

Data are expressed as mean ± standard deviation. SPSS20.0 was used to statistically compare the data from the different groups, using Duncan’s method for one-way ANOVA.

## 3. Results

### 3.1. Effect of GSP Supplementation on Rats Weight

As shown in Table 1, the body weight of rats in group B was significantly higher than that of rats in group A after four weeks of feeding (*p <* 0.05). From Table 2, the levels of blood glucose and TC in the serum of group B were significantly higher than those of group A, and the levels of HDL in the serum were significantly lower than those of group A. The differences were significant (*p <* 0.05), indicating that the obesity model of rats was established in this study and could be further studied. As shown in Table 1, after two weeks of feeding, the rats showed no significant changes in body weight between groups (*p >* 0.05). After four weeks, the body weight of rats in group B was significantly higher than that of rats in group A (*p <* 0.05). Compared with group B, the body weight of rats in groups C, D, and E showed a dose-related decrease. It was statistically significant (*p <* 0.05) and no significant difference was observed between groups C, D, and E (*p >* 0.05).

**Figure 1:**
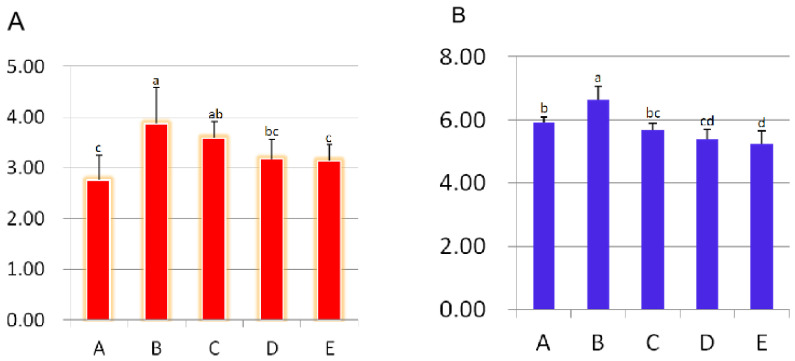
(**A**) Effects of different GSP groups on the adiposity coefficient of rats. (**B**) Effects of different GSP groups on daily weight gain of rats. Values of different groups with small superscripts mean significantly different (*p* < 0.05).

As shown in Figure 1A, after 4 weeks of feeding, the abdominal fat coefficients of rats in groups B and C were significantly higher (*p* < 0.05) than those in group A. The daily weight gain of rats in group B was increased compared to group A (*p* < 0.05). Figure 1B shows that the daily body-weight gain of rats in group B was increased (*p* < 0.05) compared with that in group A after a 4-week test period. The daily body-weight gain of rats in groups C, D, and E was significantly decreased (*p* < 0.05) compared with that in group B.

### 3.2. Effect of GSP on Blood Indicators in Rats

**Figure 2:**
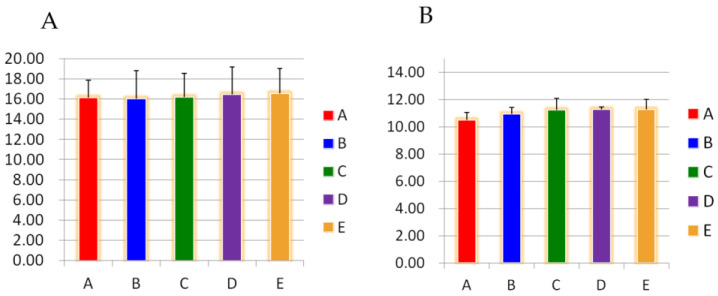
(**A**) Effects of different GSP groups on GH levels in the blood of rats. (**B**)Effects of different GSP groups on INS levels in the blood of rats. Values of the different groups with small letter superscripts mean significantly different (*p* < 0.05).

As shown in Table 2, the levels of blood glucose, TC, TG, and LDL in the serum of the GSP-treated groups were lower than those in group B after 4 weeks of feeding the rats, and the effect of GSP on the three indices of blood glucose, TC and TG was significant (*p* < 0.05). Serum HDL levels in each GSP-treated group were significantly higher than those in group B (*p* < 0.05). Figure 2A,B show that the levels of growth hormone and insulin in the serum of rats did not show significant changes between groups (*p* > 0.05).

### 3.3. Effect of GSP on Adipose Tissue Cytokine Levels

As shown in Table 3, the levels of each cytokine did not show significant changes in group B compared to group A, while the levels of ADPN did not differ significantly between the GSP treatment groups compared to group B. The levels of cAMP did not show significant changes between the groups. Levels of COX-2, IL-6, and LEP decreased in the GSP treatment groups compared with group B, with a significant decrease in COX-2 in group D (*p* < 0.05) and a significant decrease in IL-6 in groups C and E (*p* < 0.05). TNF-α levels did not change significantly between groups, and only group D was significantly higher than all other groups (*p* < 0.05).

### 3.4. Effect of GSP on Cytokine mRNA Expression in Adipose Tissue

Table 4 shows that the differences in COX-2 mRNA expression in rat adipose tissue were not significant (*p* > 0.05). The levels of LEP and TNF-α decreased in a gradient with increasing GSP concentration, and LEP decreased significantly (*p* < 0.05) in the medium and high concentration groups, and TNF-α decreased significantly (*p* < 0.05) in the high concentration group.

### 3.5. Effect of GSP on the Proliferation of 3T3-L1 Preadipocytes

**Figure 3:**
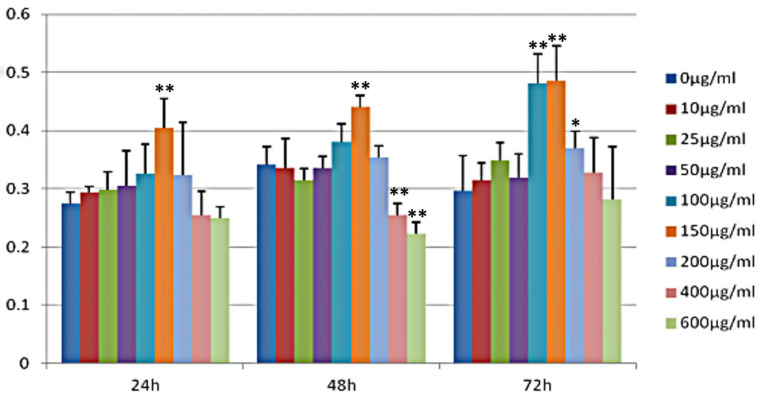
Effects of different GSP groups on proliferation of 3T3-L1 adipocytes. Values of the same time group, “*” means significant difference with control group (*p* < 0.05), “**” means (*p* < 0.01).

Figure 3 shows that the addition of different concentrations of GSP to 3T3-L1 preadipocytes had a specific effect on the proliferative activity of the cells. After 24 h, compared with the control group, the proliferative activity of the cells gradually increased with increasing GSP concentration, reaching a maximum at the concentration of 150 μg/mL, and the difference was highly significant (*p* < 0.01). At concentrations higher than 150 μg/mL, i.e., 200, 400, and 600 μg/mL, cell proliferation activity showed a decreasing trend with increasing concentrations. After 48 h, the cell proliferation activity was similar to that at 24 h and again reached a maximum at a concentration of 150 μg/mL. The effect was highly significant (*p* < 0.01) at concentrations of 200, 400, and 600 μ. Cell proliferation activity was gradually inhibited at concentrations of 200, 400, and 600 μg/mL, with 400 and 600 μg/mL significantly inhibiting cell proliferation activity (*p* < 0.01). Seventy-two hours later, 100 and 150 μg/mL concentrations significantly promoted cell proliferation activity compared to the control group (*p* < 0.01) and 200 μg/mL. However, it also significantly inhibited the proliferation activity (*p* < 0.05). (*p* < 0.05), but still showed a decreasing trend, and the higher the concentration of GSP, the more pronounced the trend.

### 3.6. Morphological Changes of 3T3-L1 Preadipocytes before and after Differentiation

**Figure 4:**
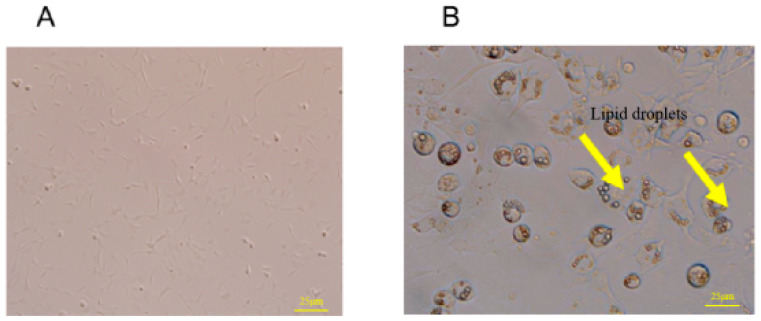
The comparison of 3T3-L1 adipocytes and differentiation of mature adipocytes (fluorescent inverted microscope, 10 times). (**A**) 3T3-L1 adipocytes. (**B**) Mature adipocytes.

The 3T3-L1 preadipocytes before induced differentiation belonged to fibroblasts and had a typical shuttle shape (Figure 4A). Adipocytes matured by induced differentiation morphologically became oval and fat droplets were observed in the cytoplasm (Figure 4B).

### 3.7. Morphological Changes in Mature Adipocytes before and after GSP Treatment

**Figure 5:**
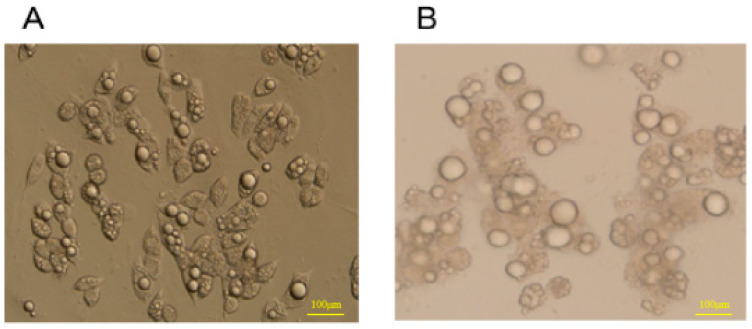
The comparison of the change of mature adipocytes before and after GSP treatment (fluorescent inverted microscope, 40 times). (**A**) Before GSP treatment. (**B**) After GSP treatment.

Before GSP treatment, mature adipocytes appeared morphologically oval with full lipid droplets in the cytoplasm (Figure 5A). However, after GSP treatment, as shown in Figure 5B, the originally full lipid droplets in the cytoplasm appeared to be crumpled.

### 3.8. Effect of GSP on Levels of Mature Adipocytokines

As shown in Table 5 and Table 6, the levels of ADP showed an increasing trend after 12, 24, and 48 h of co-incubation of different concentrations of GSP with mature adipocytes, and the levels of ADP in the 150 and 200 μg/mL concentration groups were significantly higher than those in the control group after 12 h (*p* < 0.05), but the effect gradually weakened with increasing time. After co-culture of different concentrations of GSP with mature adipocytes for 12 h, cAMP levels showed an increasing trend compared to the control group, with significant effects in the 150 and 200 μg/mL concentration groups (*p* < 0.05). After 24 h, the effect decreased. After 48 h, cAMP levels gradually decreased with increasing GSP concentration.

As can be seen from Table 7, when different concentrations of GSP were co-cultured with mature adipocytes, after 12 h the level of COX-2 gradually increased at concentrations of 0–150 μg/mL, reaching a maximum at 150 μg/mL, and the effect was significant at 12 h (*p* < 0.05). When the concentration was higher than 150 μg/mL, there was a significant decrease with a significant difference (*p* < 0.05). At 24 h, the situation was the same as at 12 h, but did not reach statistical significance. At 48 h, there were no significant changes between the groups.

After co-culture of GSP with mature adipocytes at different concentrations for 12, 24, and 48 h, no significant effects on the cytokines IL-6, LEP, and TNF-α were observed overall, but the levels of IL-6 and TNF-α decreased with increasing time. See Table 8, Table 9 and Table 10.

### 3.9. Effect of GSP on mRNA Expression of Mature Adipocyte Factors

Real-time PCR results showed that the mRNA expression of COX-2 did not show significant changes between groups after 12 and 24 h of treatment of mature adipocytes with different concentrations of GSP. Up to 48 h later, the mRNA expression level of COX-2 decreased in the GSP-treated group compared to the control group, and the difference was statistically significant (*p* < 0.05) (*p* < 0.01) in a dose–response relationship, as shown in Table 11. As shown in Table 12, GSP can significantly reduce the mRNA expression level of LEP. Compared with the control group, the mRNA expression level of LEP at 12 h in all GSP treatment groups was highly significant (*p* < 0.01) and showed a dose–response relationship. After 24 h, the mRNA expression level of LEP in all GSP treatment groups was lower than that of the control group, with the 100 and 150 μg/mL concentration groups showing a significant decrease (*p* < 0.05), and the 200 μg/mL group showing the best effect with a highly significant difference (*p* < 0.01). After 48 h, the effect of GSP was attenuated but still significantly lower in the medium and high concentration groups compared to the control group (*p* < 0.05). Table 13 shows that the effect of GSP on TNF-α was different at different time points. After 12 and 48 h, the mRNA expression level of TNF-α was highly significantly reduced in all GSP treatment groups (*p* < 0.01). At 24 h, the mRNA expression level of TNF-α was only significantly reduced in the high concentration group (*p* < 0.05). Although the other GSP treatment groups also reduced TNF-α mRNA expression, the difference did not reach statistical significance.

## 4. Discussion

### 4.1. Body Fat

The effectiveness of GSP in improving human and monogastric animal health, especially the regulation of obesity, is usually caused by abnormalities in lipid metabolism [4]. K Karthikeyan et al. [16] showed that grape seed extract has the effect of reducing body weight and improving lipid index in obese hyperlipidemic rats. However, limited information is currently available on the effect of GSP on adipocytokines. In this experiment, different doses of GSP were administered to obese rats induced by a high-fat diet. The results showed that compared to the high-fat control group, the increase in body weight of rats in each GSP treatment group was significantly inhibited (*p* < 0.05), and the abdominal fat coefficient and body weight gain were also reduced to varying degrees. The reduction in body weight gain of rats in each GSP treatment group reached a significant level (*p* < 0.05). These findings suggest that GSP has the ability to improve body mass-related indices in rats and regulate fat formation significantly.

### 4.2. Biochemical Indexes

In accordance with the improvement effect of GSP on the rat obesity, in the current study, GSP supplemented can improve the blood biochemical indexes of rats. The possible reasons may be the GSPE promoted the transition of abnormal intestinal flora structure to normal induced by high-fat diet, repaired intestinal permeability [17]. Serum cholesterol (TC), triglycerides (TG), low-density lipoprotein (LDL), and high-density lipoprotein (HDL) are common indicators of lipid metabolism in the body. Some studies have shown that GSP can regulate blood lipids and reduce serum levels of TC and TG. The results of the present experiment showed that GSP had a significant effect on reducing serum TC levels, which is consistent with the findings of Sylwia et al. [18]. GSP treatment reduced serum TG and LDL in high-fat rats, and the effect of TG reduction was significant (*p* < 0.05), which supports the findings of Song, C [19]. The level of serum HDL was increased to varying degrees and reached statistical significance (*p* < 0.05). Guerrero, L [20] also found that GSP reduced serum LDL and TG and increased HDL levels. In addition, in vivo experimental studies found that GSP has potent effects on regulating lipid metabolism, improving blood lipids, promoting cholesterol reversal, and accelerating lipid and cholesterol excretion [16]. Mei Yin’s results are similar to those in this experiment [21].

Since obesity is usually accompanied by abnormal glucose metabolism, this experiment found that the GSP-treated group significantly reduced the blood glucose level in the serum of obese rats (*p* < 0.05) as well as increased the insulin level compared with the high-fat material control group, indicating that GSP has the effect of ameliorating hyperglycemia in obese rats. Growth hormone can accelerate lipolysis by inhibiting sugar consumption and shifting the energy source from glucose metabolism to fat metabolism. In this experiment, the GSP-treated group was able to increase serum growth hormone levels compared to the control group, thereby accelerating lipolysis and achieving the effect of reducing fat deposition in rats. Grape seed proanthocyanidins could inhibit fat deposition in obese rats and effectively improve blood lipid index, thus regulating glucolipid metabolism in obese rats. The rats showed no diarrhea or appetite suppression during the study period. This study provides further evidence for the effect of GSP in reducing serum lipids in the population.

Human lipocalin is localized at 3q27, a locus closely associated with type 2 diabetes and its metabolic syndrome, hence its antidiabetic properties. Type 2 diabetic patients are characterized by a significant decrease in their serum lipocalin levels and a significant increase as their body weight decreases. One study [22] found that plasma ADPN levels were significantly negatively correlated with serum TG and atherosclerotic index (TC/HDL), whereas they were significantly positively correlated with serum HDL levels. Various factors can regulate the secretion and level of ADPN, such as adipocytokines, as resistin, tumor necrosis factor-α and IL-6 have been reported to downregulate ADPN expression [23]. In this study, we found that after feeding rats with different concentrations of GSP, the GSP-treated group could increase the level of ADPN in adipose tissue compared to the high-fat diet control group, which did not reach statistical significance but provided a basis that GSP could increase the level of ADPN in adipose tissue.

Serum levels of IL-6 have been shown to be elevated in overweight and obese individuals [24]. The secretion of IL-6 in adipose tissue is also influenced by various factors, and in this study the GSP-treated group had a significant reduction in IL-6 levels compared to the high-fat control group (*p* < 0.05), indicating that GSP has the effect of reducing IL-6 levels in adipose tissue and that the effects of ADPN and IL-6 are antagonistic to each other.

COX-2 is an important inflammatory cell mediator and Mudit et al. [25] found that GSP inhibited the growth and accelerated the apoptosis of non-small cell lung cancer cells (A549, H1299, H460, H226, and H157) by suppressing the overexpression of COX-2, prostaglandin E2, and prostaglandin E2 receptors (EP1 and EP4). In the present study, GSP treatment significantly reduced COX-2 levels compared to the high-fat control group, and the effect was better in the mid-dose group (*p* < 0.05). Moreover, at the molecular level, GSP reduced COX-2 mRNA expression, with a significant effect at low doses (*p* < 0.05). This indicates that GSP has the ability to reduce COX-2 levels in adipose tissue, resulting in a positive correlation between COX-2 levels and IL-6 levels in adipose tissue, a result consistent with the conclusion of Syed et al. [26] that COX-2 is positively correlated with body weight, visceral fat weight, and the inflammatory factors IL-6 and TNF-α. In this experiment, with the increase in GSP concentration, the amount of body fat in obese rats gradually decreased, and the LEP level in each treatment group of GSP did not show significant changes compared with the high-fat material control group, but the middle and high dose groups showed a decreasing trend, and perhaps the decreasing trend will be significantly different at a higher dose, and this result is basically consistent with the conclusion of Mi Liu’s experiment. In the fluorescence quantification test, the mRNA expression of LEP was significantly reduced in the medium-high dose group compared with the high-fat control group (*p* < 0.05), which is further evidence of the function of GSP in reducing body fat.

The levels of TNF-α did not change significantly between groups in this study, and TNF-α in the medium-dose group was also significantly higher than in the other groups (*p* < 0.05). This result is not consistent with the mechanism of action of TNF-α, and the analysis may be due to the fact that the dose and duration of action of GSP used in this trial are not sufficient, so the effect on the large amount of TNF-α produced by obesity is not obvious. However, results at the molecular level showed that the high dose of GSP significantly reduced the mRNA expression of TNF-α compared with the high-fat control group (*p* < 0.05). The reason for this result may be that the effect of GSP is more easily manifested at the molecular level, which is more sensitive. In addition, although the adipose tissues used in the ELISA and molecular assays are from the same rat peritoneal cavity, they may give different results due to their slightly different locations. This is because there are differences in adipose tissue characteristics between different species, between different fat depots of the same individual, or even between different parts of the same fat depot [12].

In order to maintain normal cellular metabolism in the body, it is important to maintain a constant ratio of cAMP and cGMP concentrations in tissues and plasma to maintain normal cellular metabolism in the body. In this experiment, the changes caused by obesity in rats were not reflected in the levels of cAMP in all groups. This is because the lipid abnormalities associated with obesity may only affect the levels of cGMP, thus disturbing the balance between the concentrations of cAMP and cGMP.

Adipose tissue is an important regulator of the body’s energy storage, endocrine, immune, and inflammatory responses, and understanding adipocytokine function is related to the study of adipocytokines. There are several ways in which adipocytokines regulate energy metabolism in the body, and the concentration levels of many adipocytokines are related to the amount of body fat. Therefore, a deeper understanding of the biosynthetic pathways and mechanisms of action of these cytokines at the molecular level may open up new avenues for the treatment of obesity, insulin resistance, and type 2 diabetes [27,28].

### 4.3. Adipocytokines

The results of this experimental study showed that within a certain concentration range (≤150 μg/mL), GSP can promote the proliferation of 3T3-L1 preadipocytes with a time–dose dependence, and when the concentration is too high (>150 μg/mL), GSP inhibits the proliferation of 3T3-L1 preadipocytes, and the higher the concentration, the more obvious the inhibitory effect. This suggests that GSP has the same dual effect on the proliferation of 3T3-L1 preadipocytes, and whether its mechanism of action is the same as that of sea buckthorn fatty acid remains to be verified.

The differentiation of precursor adipocytes into mature adipocytes requires growth inhibition, clonal proliferation, and a series of changes in gene expression, with a gradual increase in the number of intracellular lipid droplets and the pooling of small lipid droplets into large lipid droplets until they fill most of the adipocyte [29]. In this experiment, in terms of adipocyte differentiation, the addition of different doses of GSP resulted in the crumpling of lipid droplets in adipocytes and reduced fat accumulation. Similarly, Liu Liu Ming et al. [17], in investigating the lipid-regulating mechanism of total hawthorn leaf flavonoids, found that total hawthorn leaf flavonoids played a certain inhibitory role in the differentiation of preadipocytes into mature adipocytes and the formation of intracellular fat.

In this experiment, by detecting the levels of cytokines in the cell culture supernatant, we found that GSP treatment also increased the levels of ADPN secreted by mature adipocytes at different time periods compared to the control group. For the two indicators, cAMP and COX-2, within a certain concentration range (≤150 μg/mL), GSP could increase their secretion levels, and when the concentration was too high, there was a decreasing trend, indicating that the higher the concentration of GSP, the better the inhibitory effect on adipocytes. However, GSP treatment had no significant effect on the levels of IL-6, LEP, and TNF-alpha secreted by mature adipocytes. This analysis may be due to the fact that GSP has a strong inhibitory effect on the secretion of these cytokines in cells, making them difficult to detect in the culture medium. In addition, the cell source may also be one of the reasons for this result, since adipose tissue is distributed in each independent fat depot of the body, and each fat depot has its own specificity in the expression and secretion of adipocytokines. “Adipose tissue is not composed of pure adipocytes, but also contains a large number of non-adipocytes, which play an important role in the secretion of adipocytokines and in the response of adipose tissue to adipocytokines and other signals. Primary culture of human adipose tissue has shown that, with the exception of adiponectin and leptin, which are specifically secreted by adipose tissue, more than 90% of the rest is produced by non-adipocytes, and that visceral adipose tissue releases more vascular endothelial growth factor (VEGF) and IL-6 than abdominal subcutaneous adipose tissue, a difference attributed to non-adipocytes [30]. In the present experiment, after collecting the cells, the mRNA expression of each cytokine in the cells could be detected, and the mRNA expression levels of COX-2, LEP, and TNF-α were significantly lower in the GSP-treated group than in the control group, further indicating that GSP has some inhibitory effect on mature adipocytes.

The dual effect of GSP on the proliferation of 3T3-L1 preadipocytes and its inhibitory effect on mature adipocytes may be the intrinsic mechanism of its inhibition of fat deposition. However, this result may be different or even opposite to other flavonoids, and the analysis may be caused by the different sources and concentrations of flavonoid extracts and the duration of action. Flavonoids contain a wide range of compounds that modulate multiple mechanisms in organisms and also demonstrate the multi-target effect of herbal drugs on organisms.

## 5. Conclusions

Grape seed proanthocyanidins (GSP), a natural plant extract, comprises flavanol monomers and their polymeric polyphenolic compounds. This study investigated the effects of GSP on lipid metabolism and adipocytokines in obese rats in vitro and in vivo. The results demonstrated that GSP could improve blood lipid indexes, regulate glucolipid metabolism, and affect the expression levels of lipid metabolism-related cytokines and their mRNA in obese rats.

In vivo tests revealed that GSP inhibited the increase in body weight, daily weight gain, and abdominal fat coefficient, and significantly improved blood glucose, TC, TG, HDL, LDL, insulin, and growth hormone levels compared to the high-fat material control group. The high-fat material + GSP 100 mg/d group showed the best overall effect. Moreover, the moderate amount of GSP demonstrated the tendency to increase the level of cytokine ADPN in adipose tissue, decrease the level of COX-2, IL-6, and LEP, and reduce the mRNA expression of COX-2, LEP, and TNF-α, providing some theoretical basis for GSP to regulate glucolipid metabolism in obese rats.

In vitro tests demonstrated that GSP had an inhibitory effect on the proliferation of 3T3-L1 preadipocytes at high concentrations (>150 μg/mL) compared to the control. Additionally, GSP caused the crumpling of lipid droplets of mature adipocytes and increased secretion of ADPN in adipocytes at concentrations ≤150 μg/mL while increasing secretion of cAMP and COX-2. In contrast, it decreased secretion of cAMP and COX-2 at concentrations of 200 μg/mL. Compared with the control group, GSP reduced the mRNA expression of COX-2, LEP, and TNF-α in adipocytes at different time periods, and the GSP group at 200 μg/mL had the best effect after 48 h of action.

In summary, GSP at 100 mg/d can effectively control the body weight of obese rats. Furthermore, the addition of GSP at 200 μg/mL can crumple the adipocyte in vitro, reduce the mRNA expression of COX-2, LEP, and TNF-α in adipocytes in vitro, and increase secretion of cAMP and COX-2 at low concentrations while decreasing it at high concentrations. Additionally, GSP can improve blood lipid indexes and affect the expression levels of lipid metabolism-related cytokines and their mRNA in obese rats. These findings suggest that GSP has the potential to regulate glucolipid metabolism and may be a promising candidate for the prevention and treatment of obesity-related diseases. However, further studies are needed to explore the exact mechanisms of action of GSP and determine optimal dosages for human use.

## Figures and Tables

**Table 1:** Effects of different GSP groups on weight of rats (g).

	0 w	2 w	4 w
A	116.87 ± 7.95	183.27 ± 14.91	282.32 ± 10.83 ^b^
B	113.87 ± 8.99	194.25 ± 8.71	299.09 ± 15.98 ^a^
C	111.38 ± 7.81	184.52 ± 15.25	269.99 ± 11.63 ^bc^
D	109.07 ± 8.29	177.72 ± 16.99	259.69 ± 13.81 ^c^
E	109.10 ± 6.52	180.96 ± 13.41	255.44 ± 15.83 ^c^

Values of the same indexes in the same column with small letter superscripts mean significantly different (*p* < 0.05).

**Table 2:** Effects of different GSP groups on blood glucose, TC, TG, HDL, and LDL levels in the blood of rats.

	Glu (mmol/L)	TC (mmol/L)	TG (mmol/L)	HDL (mmol/L)	LDL (mmol/L)
A	9.70 ± 0.78 ^b^	1.43 ± 0.33 ^b^	0.63 ± 0.17 ^ab^	165.42 ± 11.26 ^ab^	55.22 ± 2.75
B	14.08 ± 3.11 ^a^	2.27 ± 0.42 ^a^	0.78 ± 0.11 ^a^	160.41 ± 3.88 ^b^	58.17 ± 3.21
C	10.09 ± 4.14 ^b^	1.85 ± 0.17 ^ab^	0.73 ± 0.13 ^ab^	172.56 ± 6.00 ^a^	55.37 ± 0.83
D	9.06 ± 2.37 ^b^	1.83 ± 0.39 ^ab^	0.58 ± 0.10 ^b^	174.89 ± 12.03 ^a^	57.04 ± 1.70
E	7.82 ± 3.03 ^b^	1.75 ± 0.58 ^b^	0.70 ± 0.12 ^ab^	176.05 ± 11.45 ^a^	56.64 ± 2.76

Values of the same indexes in the same column with small letter superscripts mean significantly different (*p* < 0.05).

**Table 3:** Effects of different GSP groups on the levels of cytokines in adipose tissue of rats.

	A	B	C	D	E
ADPN (μg/L)	51.35 ± 7.95	51.01 ± 8.31	55.17 ± 8.77	53.96 ± 6.68	54.66 ± 9.04
cAMP (nmol/L)	7.16 ± 0.46	7.00 ± 0.66	7.01 ± 0.56	6.97 ± 0.44	6.96 ± 0.33
COX-2 (pg/mL)	110.77 ± 12.59 ^ab^	114.45 ± 13.00 ^a^	106.21 ± 9.71 ^ab^	98.69 ± 10.77 ^b^	103.51 ± 12.88 ^ab^
IL-6 (ng/L)	112.45 ± 7.53 ^ab^	114.84 ± 5.13 ^a^	106.27 ± 8.96 ^b^	106.89 ± 6.73 ^ab^	106.21 ± 5.93 ^b^
LEP (μg/L)	4.53 ± 0.24	4.58 ± 0.51	4.61 ± 0.24	4.41 ± 0.38	4.42 ± 0.19
TNF-α (ng/L)	208.55 ± 5.78 ^ab^	205.26 ± 5.71 ^b^	204.73 ± 6.03 ^b^	214.56 ± 6.40 ^a^	205.61 ± 5.19 ^b^

Values of the same indexes in the same line with small letter superscripts mean significantly different (*p* < 0.05).

**Table 4:** Effects of different GSP groups on COX-2, LEP, TNF-α mRNA expression in adipose tissue of rats.

	COX-2 (pg/mL)	LEP (μg/L)	TNF-α (ng/L)
A	8.37 ± 1.41	5.53 ± 0.57 ^b^	6.85 ± 0.80 ^b^
B	10.24 ± 1.14	7.64 ± 1.01 ^a^	9.27 ± 0.24 ^a^
C	7.53 ± 0.77	7.41 ± 1.05 ^a^	9.57 ± 0.86 ^a^
D	7.97 ± 0.68	5.69 ± 1.05 ^b^	8.16 ± 0.95 ^ab^
E	8.27 ± 1.18	4.93 ± 1.04 ^b^	6.66 ± 0.52 ^b^

Values of the same indexes in the same line with small letter superscripts mean significantly different (*p* < 0.05).

**Table 5:** Effect of different concentrations of GSP on ADP levels in mature adipocytes.

	(μg/mL)		Time	
		12 h	24 h	48 h
ADP (μg/L)	0	35.54 ± 3.54 ^b^	36.76 ± 3.81	29.24 ± 3.80
	100	42.40 ± 2.30 ^ab^	36.85 ± 5.02	24.09 ± 3.72
	150	47.87 ± 3.44 ^a^	39.80 ± 4.10	29.72 ± 4.41
	200	50.73 ± 5.04 ^a^	42.79 ± 5.56	33.40 ± 3.46

Values of the same indexes in the same column with small letter superscripts mean significantly different (*p* < 0.05).

**Table 6:** Effect of different concentrations of GSP on cAMP levels in mature adipocytes.

	(μg/mL)		Time	
		12 h	24 h	48 h
cAMP (nmol/L)	0	2.02 ± 0.22 ^b^	2.41 ± 0.56	2.13 ± 0.05
	100	2.20 ± 0.51 ^ab^	2.29 ± 0.43	2.03 ± 0.53
	150	3.06 ± 0.28 ^a^	2.86 ± 0.33	1.81 ± 0.58
	200	3.03 ± 0.17 ^a^	2.56 ± 0.42	1.61 ± 0.56

Values of the same indexes in the same column with small letter superscripts mean significantly different (*p* < 0.05).

**Table 7:** Effect of different concentrations of GSP on COX-2 levels in mature adipocytes.

	(μg/mL)		Time	
		12 h	24 h	48 h
COX-2 (pg/mL)	0	1606.46 ± 234.82 ^b^	1747.08 ± 181.39	1479.38 ± 146.28
	100	2027.29 ± 145.46 ^ab^	1850.21 ± 237.05	1709.58 ± 282.46
	150	2088.75 ± 256.12 ^a^	2047.08 ± 310.50	1593.96 ± 174.67
	200	1970.00 ± 224.67 ^ab^	1842.92 ± 145.11	1682.50 ± 162.65

Values of the same indexes in the same column with small letter superscripts mean significantly different (*p* < 0.05).

**Table 8:** Effect of different concentrations of GSP on IL-6 levels in mature adipocytes.

	(μg/mL)		Time	
		12 h	24 h	48 h
IL-6 (ng/L)	0	49.11 ± 8.75	42.75 ± 3.19	40.50 ± 4.44
	100	43.03 ± 1.58	38.17 ± 4.33	38.88 ± 0.95
	150	52.78 ± 7.84	51.44 ± 7.28	47.25 ± 6.38
	200	49.82 ± 2.68	42.63 ± 3.62	45.00 ± 2.95

**Table 9:** Effect of different concentrations of GSP on LEP levels in mature adipocytes.

	(μg/mL)		Time	
		12 h	24 h	48 h
LEP (μg/L)	0	4.68 ± 0.87	4.59 ± 0.44	4.62 ± 0.49
	100	4.27 ± 0.26	4.31 ± 0.11	4.76 ± 0.38
	150	4.69 ± 0.57	4.19 ± 0.57	5.06 ± 0.57
	200	4.71 ± 0.30	4.39 ± 0.35	4.81 ± 0.26

**Table 10:** Effect of different concentrations of GSP on TNF-α levels in mature adipocytes.

	(μg/mL)		Time	
		12 h	24 h	48 h
TNF-α (ng/L)	0	460.39 ± 46.43	445.85 ± 37.39	438.29 ± 35.47
	100	433.45 ± 32.50	438.30 ± 16.37	402.44 ± 22.67
	150	483.45 ± 34.49	441.01 ± 24.68	432.29 ± 23.85
	200	474.34 ± 19.37	458.64 ± 6.81	442.75 ± 47.96

**Table 11:** Effect of different concentrations of GSP on COX-2 mRNA expression in mature adipocytes.

	(μg/mL)		Time	
		12 h	24 h	48 h
COX-2 (pg/mL)	0	7.61 ± 1.87	6.27 ± 1.57	9.54 ± 0.61
	100	5.40 ± 1.69	8.18 ± 1.59	6.64 ± 1.59 *
	150	7.19 ± 1.22	7.39 ± 1.46	5.01 ± 0.04 **
	200	6.40 ± 1.07	7.08 ± 1.18	2.51 ± 0.43 **

Values of the same time group, “*” means significant difference with control group (*p* < 0.05), “**” means (*p* < 0.01).

**Table 12:** Effect of different concentrations of GSP on LEP mRNA expression in mature adipocytes.

	(μg/mL)		Time	
		12 h	24 h	48 h
LEP (μg/L)	0	14.47 ± 0.14	16.13 ± 3.02	11.19 ± 1.43
	100	5.96 ± 1.89 **	11.08 ± 2.70 *	9.07 ± 2.41
	150	8.69 ± 0.72 **	11.11 ± 3.20 *	4.12 ± 1.53 *
	200	3.34 ± 0.00 **	7.90 ± 1.05 **	1.96 ± 0.85 **

Values of the same time group, “*” means significant difference with control group (*p* < 0.05), “**”means (*p* < 0.01).

**Table 13:** Effect of different concentrations of GSP on TNF-α mRNA expression in mature adipocytes.

	(μg/mL)		Time	
		12 h	24 h	48 h
TNF-α (ng/L)	0	14.14 ± 0.73	16.44 ± 3.08	8.17 ± 1.74
	100	7.79 ± 1.22 **	11.74 ± 2.51	5.03 ± 1.77 **
	150	10.11 ± 1.57 **	11.65 ± 1.19	4.80 ± 1.63 **
	200	6.96 ± 1.03 **	8.42 ± 1.13 *	0.43 ± 0.36 **

Values of the same time group, “*” means significant difference with control group (*p* < 0.05), “**” means (*p* < 0.01).

## Data Availability

All data are contained within the article.
